# Pan‐risk factor for a comprehensive cardiovascular health management

**DOI:** 10.1111/1753-0407.13258

**Published:** 2022-02-28

**Authors:** Ruizhi Zheng, Yu Xu, Mian Li, Jieli Lu, Min Xu, Tiange Wang, Zhiyun Zhao, Shuangyuan Wang, Hong Lin, Xiaoyun Zhang, Yufang Bi, Weiqing Wang, Guang Ning

**Affiliations:** ^1^ Department of Endocrine and Metabolic Diseases, Shanghai Institute of Endocrine and Metabolic Diseases Ruijin Hospital, Shanghai Jiao Tong University School of Medicine Shanghai China; ^2^ Shanghai National Clinical Research Center for Metabolic Diseases, Key Laboratory for Endocrine and Metabolic Diseases of the National Health Commission of the PR China, Shanghai Key Laboratory for Endocrine Tumor State Key Laboratory of Medical Genomics, Ruijin Hospital, Shanghai Jiao Tong University School of Medicine Shanghai China

**Keywords:** cardiovascular diseases, primary prevention, risk factors, 心血管疾病, 危险因素, 一级预防

## Abstract

Cardiovascular diseases (CVDs) have become the leading cause of death in China. CVDs are mainly caused by multiple well‐known modifiable risk factors that are affected by socioeconomic and environmental determinants, lifestyle and behavioral choices, and familial and genetic predispositions. With more risk factors proved to be associated with CVD occurrence, the concept “pan‐risk factor” is proposed in this review to indicate all discovered and yet‐to‐be‐discovered CVD risk factors for comprehensive primary prevention of CVD. Recognizing more factors and their roles in CVD development and progression is the first step in reducing the ever‐increasing burden of CVD. This review is an overview of the pan‐risk factor whose associations with CVD outcomes have been established. Along with the accumulation of scientific evidence, an increasing number of risk factors will be discovered and included in the list of pan‐risk factors.

## Graphical abstract

**Highlights**

- Management and intervention on cardiovascular risk factors are the primary prevention strategy to reduce the burden.
- Promotion of cardiovascular health is particularly urgent for China because of the ever‐rising prevalence of these risk factors.
- The concept “pan‐risk factor” is proposed to indicate all discovered and yet‐to‐be‐discovered cardiovascular risk factors for a comprehensive and exhaustive primary prevention of cardiovascular diseases.

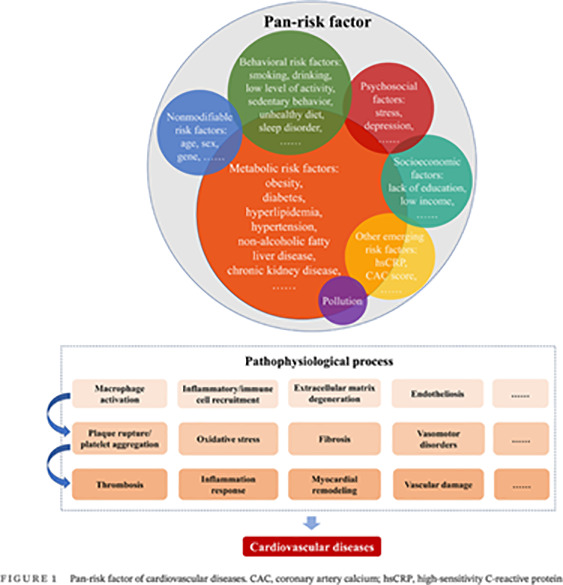

## 摘要

在中国，心血管疾病(CVD)已成为主要的死亡原因。心血管疾病主要是由多种众所周知的可改变的危险因素引起，这些危险因素受社会经济和环境因素, 生活方式, 行为选择以及家庭和遗传易感性的影响。随着越来越多的危险因素被证实与心血管疾病的发生有关，本文提出了“泛危险因素”的概念和术语，指所有已发现和尚未发现的心血管疾病危险因素，用于心血管疾病的一级预防。认识到更多的因素及其在心血管疾病进展中的作用，是减轻日益加重的心血管疾病负担的第一步。这篇综述是对“泛危险因素”的概述，这些因素与心血管疾病预后的关系已经确定。随着科学证据的积累，越来越多的风险因素会被发现，并被列入“泛危险因素”的名单中。

## 1 INTRODUCTION

In the past 3 decades, China has experienced great progress in health promotion.^1^ However, rapidly increasing income results in a dramatic change of lifestyle. These changes have been accompanied by increasing morbidity of noncommunicable diseases.^1^ Consequently, cardiovascular diseases (CVDs) gradually turn into the principal reason for death in China.^1^ CVDs can be triggered by multiple risk factors: 63% of CVD events are accounted for by physical inactivity, unhealthy diet, smoking, and metabolic factors.^2^, ^3^ The early‐life exposure to famine or obesity might also relate to an increased chance of suffering from CVD in later life.^4^, ^5^ With more risk factors proved to be associated with CVD, we propose the term “pan‐risk factor” to indicate all CVD risk factors. Knowledge of these risk factors provides a basis for developing preventive strategies for CVD management.

## 2 NONMODIFIABLE RISK FACTORS

### 2.1 Age

Life expectancy has risen remarkably during the past half‐century. In an aging society, people have longer exposure to risk factors. Age plays a vital part in the deterioration of both physiological and cardiovascular functionality, which includes changes of the contractile and conduction systems of the heart, structural and electrical conduction heterogeneity, supraventricular and ventricular arrhythmias.^6^ The American Heart Association (AHA) demonstrates that the incidence of stroke is less than 3% in people aged 20–59 years, it increases to 8% among those aged 60–79 years, and reaches 13%–17% in those aged over 80 years.^7^ Given that the number of older patients will continue to increase, it is important to test treatment strategies in older patients, especially for those aged over 75, in future studies.

### 2.2 Sex

Sex differences in CVD risk may have originated from an interaction between the gene with androgen receptor located on the X chromosome and sex hormones that provide the basis for sex differences in cardiovascular regulatory mechanisms in the lifespan.^8^ The Y chromosome is one of the strongest genetic determinants of coronary artery disease (CAD).^9^, ^10^ Even in the presence of similar CVD risk factors for both men and women, compared with men, women suffer from CVD 7 to 10 years later, which has been largely ascribed to the protective effects of estrogen before menopause.^11^ The marked reduction in estrogen levels in and after menopause may explain the delayed clinical onset of CVD in women. However, there remain several clinical conditions leading to the increased, and even early‐onset, CVD risk unique to women, including polycystic ovary syndrome, gestational diabetes, and early menopause.^11^ Sex differences are also found in the clinical manifestation of CVD and relevant risk factors, including ischemic heart disease (IHD), heart failure, pressure overload, hypertension, and arrhythmia.^12^, ^13^ A deep understanding of sex‐specific cardiovascular pathological mechanisms and their underlying complex mechanisms and pathways is useful for developing treatment strategies.

### 2.3 Gene

Genetic background greatly affects CVD risk. The inheritance of CAD from genetic variation is estimated to be 40% to 60%.^14^ The common variants account for about 38% of the heritability.^15^ Monogenic CVD including inherited cardiomyopathies, rhythm disorders, and vascular disorders is more likely to be caused by rare DNA variants by which large effects are triggered. On the contrary, hundreds of common genetic variants that happened occasionally throughout the genome have been discovered to be associated with increased CVD risk. However, it is difficult to recognize the genetic mechanism of complex morbidities because their pathogenic origins are the consequence of a variety of genetic effects and environmental effects. Many genes are found to be associated with CVD, including *LDLR* (low‐density lipoprotein [LDL] receptor), *LPA* [lipoprotein(a), or Lp(a)], *LPL* (lipoprotein lipase), *APOA5* (apolipoprotein A‐V), *APOC3* (apolipoprotein C‐III), *ANGPTL4* (angiopoietin‐like 4), and *ASGR1* (asialoglycoprotein receptor 1).^16^, ^17^

## 3 MODIFIABLE RISK FACTORS

### 3.1 Lifestyle risk factors

#### 3.1.1 Smoking

Tobacco use produces more than 7000 chemicals causing innumerable impacts on the deterioration of the cardiovascular system accelerating the pathogenesis of CVD. To be concrete, the toxic chemicals inhaled with smoking including nicotine, carbon monoxide, polycyclic aromatic hydrocarbons, and heavy metals exacerbate the condition of impaired vascular endothelium, blood lipids disorders, insulin resistance, and clotting factors disorders causing thrombus and atherosclerosis, activating inflammatory pathways and increasing demand for the diminishing supply of myocardial oxygen.^18^ Even a small dose of tobacco smoking also increases the risk of suffering CVD in later life (eg, even smoking <5 cigarettes per day).^19^ Passive exposure to cigarette smoking also damages cardiovascular health. Studies demonstrate that early exposure to cigarette smoking during fetal development causes significantly lower birth weights but a higher possibility of obesity and hypertension in later life.^20^, ^21^ There has been a substantial rise in the use of electronic cigarettes (e‐cigarettes) in place of conventional smoking. The aerosol produced by the e‐cigarette also contains nicotine and toxic and cancer‐causing chemicals. The cardiovascular harm from e‐cigarette use might occur because of increased risk of thrombosis, atherosclerosis, and enhanced sympathetic activities.^22^ Evidence from ecological studies confirmed that living under a smoke‐free atmosphere is closely associated with a lower risk of CVD.^23^, ^24^ Rigorous tobacco control, for instance by strong tobacco taxation policy and smoke‐free legislation, would prevent the rising trend of tobacco consumption and hence reduce CVD risk related to smoking.

#### 3.1.2 Alcohol drinking

There is still controversy on the protective effects of low or moderate drinking on CVD risk. Low‐risk limits for alcohol consumption vary substantially across different recommendations. A combined analysis of 83 prospective studies summarized that the threshold for the lowest risk of all‐cause mortality was about 100 g/week, but no clear thresholds below for lower CVD risk.^25^ Several mechanisms had been proposed to explain the beneficial effects of moderate alcohol consumption on the cardiovascular function, which include an increase in high‐density lipoprotein (HDL)‐cholesterol and insulin sensitivity, enhanced anti‐inflammatory effect, increased adiponectin, and decrease in platelet aggregation and coagulation.^26^ Red wine contains high concentrations of polyphenolic compounds such as flavonoids, resveratrol, and polymeric tannins. The phenolic compounds in red wine have desirable biological actions, including cardiovascular protection effects.^27^ The 2‐yearCArdiovaSCulAr Diabetes & Ethanol (CASCADE) trial had evaluated the effects of moderate wine consumption on cardiometabolic risk factors in adults with type 2 diabetes.^28^ The study found that initiating moderate wine intake (150 ml), especially red wine, among well‐controlled diabetics in the context of a healthy diet is safe and modestly decreases cardiometabolic risk.^28^ However, one study using Mendelian randomization analysis proved that the apparently protective effects of moderate alcohol intake against stroke are largely noncausal. Alcohol intake uniformly increases blood pressure and stroke risk.^29^ Taking 280 g per week of alcohol increases systolic blood pressure by about 5 mm Hg, which is connected with an increase of 15% in the risk of IHD and ischemic stroke and about 30% in the risk of intracerebral hemorrhage among Chinese people.^30^ In China, there is an increasing trend in the prevalence of alcohol dependence from 0.02% to 0.68% between the 1980s and 1990s, following the almost three‐fold increase of alcohol consumption per capita from 2.5 L in 1978 to 7.2 L in 2016.^31^ The adverse effects of alcohol drinking on the high burden of CVD incidence should not be ignored in China.

#### 3.1.3 Sedentary behavior and physical inactivity

Sedentary behavior independently increases the risk of chronic noncommunicable disease. However, the evidence is insufficient to inform public health guidance.^32^ An experimental study using hindlimb suspension (unloading) in a rat model to mimic human sedentary behavior, a decrease in lipoprotein lipase activity, triglyceride uptake into skeletal muscle, and HDL‐cholesterol level occurred during a day.^33^ A meta‐analysis reported a significant hazard risk associated with sedentary behavior in groups with low levels of physical activity but a nonsignificant hazard risk in groups with high levels of physical activity.^34^ Ekelund et al had examined the joint impacts of sedentary behavior and physical activity on CVD mortality. They demonstrated that moderate‐to‐vigorous physical activity at every level of sitting (<2, 2–5.9, 6–8, and > 8 h per day) was positively associated with lower CVD mortality.^35^

Evidence from rodent models showed that a high level of physical activity enhances calcium handling through the sarcoendoplasmic reticulum calcium transport ATPase.^36^ Regular physical activity also benefits the peripheral vasculature by reducing mitochondrial reactive oxygen species production, enhancing cellular antioxidant defense proteins.^37^ Decreased physical activity is regarded as the fourth risk factor for coronary artery disease in AHA guidelines.^38^, ^39^ From 2000 to 2014 in China, the percentage of people achieving the minimum recommendation goals by 2019 American College of Cardiology/AHA guideline slowly increased over time (from 17.2% in 2000 to 22.8% in 2014).^40^ Nationwide programs are imperative to promote more physical activity and healthier lifestyles to keep fit in the Chinese population.

#### 3.1.4 Unhealthy diet

##### Nutrients

The importance of nutrition in preventing CVD is well established in many studies about macronutrients (carbohydrates, fats, proteins, macrominerals, and water), micronutrients (vitamins and other minerals), and other nutrients (fiber and dietary supplements). Because of the difficulties in precisely assessing the composition of the diet, observational studies always obtain opposite conclusions in the association between some nutrients and CVD. For example, the Prospective Urban Rural Epidemiology (PURE) study recently challenged contemporary well‐known thought, suggesting that compared with intake of refined carbohydrates, saturated and unsaturated fat intake are related to a lower risk of stroke and mortality.^41^ Ho et al observed that fiber, protein, and polyunsaturated fatty acids intake are nonlinearly associated with incident CVD.^42^ In contrast, a diet that is composed of total carbohydrate and total fat (including monounsaturated fatty acids) is linearly associated with incident CVD risk.^43^ Dietary sodium intake is highly correlated with hypertension, subsequently putting individuals at risk of CVD and premature death. However, a recent study found that sodium intake is inversely associated with life expectancy and positively related to lower all‐cause mortality.^43^ Recently, a randomized trial demonstrated the beneficial effects of salt substitutes with reduced sodium levels and increased potassium levels among persons who had a history of stroke or were over 60 years of age and had hypertension.^44^ After 4.74 years of follow‐up, the rates of stroke, major cardiovascular events, and death from any cause were lower with the salt substitute than with regular salt.^44^ Vitamin D deficiency is a marker of increased cardiovascular risk related to cardiovascular events.^45^ However, meta‐analyses show that oral vitamin D supplementation does not protect against cardiovascular events.^46^

##### Food

An unhealthy diet exacerbates the burden of metabolic disease, which leads to CVD morbidity. A healthy diet consists not only of high consumption of nonstarchy vegetables, fruits, whole grains, and legumes but also moderate consumption of nuts, seafood, lean meats, low‐fat dairy products, and vegetable oil in balance. During the past decades the consumption of ultraprocessed foods has increased substantially around the world. A prospective cohort study found that an absolute increment of 10 in the percentage of ultraprocessed foods in the diet was associated with 11% ~ 13% statistically significant increase in the rates of overall cardiovascular, coronary heart, and cerebrovascular disease.^47^ Another study also observed that ultraprocessed meat intake was associated with increased risk of hard and overall CVD.^48^ Evidence suggests that regular intake of tea and its active ingredients including epigallocatechin gallate may bring benefits to body health, the mechanisms may include reducing oxidative stress and inflammation response, elevating nitric oxide bioavailability to improve vascular permeability, and lowering blood pressure (BP).^49^ Coffee consisted of compounds including caffeine, anti‐ or pro‐oxidant substance have functions on vascular regulation. These compounds are speculated to be complexly intertwined to play a role in the regulation of endothelial function. Epidemiological studies have shown a J‐shaped curve, which means that regular coffee intake has an inverse or no association with myocardial infarction, stroke, and CVD.^50^, ^51^ Besides, soft drink consumption is a widely acknowledged adverse health behavior. Several prospective cohort studies all confirmed an increased risk of CVD related to soft drink consumption.^52^

##### Overnutrition and malnutrition

Overnutrition, which is characterized by excessive nutrient and energy intake, results in obesity, a growing global health threat in modern times. Malnutrition comes into clinical manifestation when the body cannot use enough energy, protein, vitamins, and other nutrients.^53^ The common causes include poverty, pregnancy or rapid growth, alcohol addiction, living alone, being socially isolated, or discharged from the hospital. Epidemiological studies have repeatedly demonstrated that childhood exposure to famine or severe malnutrition results in long‐term risk of CVD.^54^ Adults with chronic exposure to malnutrition are also associated with a high risk of acute CVD events.^55^

##### Dietary pattern

Many societies have provided several healthy diet patterns focusing on CVD prevention and weight loss, such as the Dietary Approaches to Stop Hypertension (DASH), Mediterranean diet, ketogenic diet, low carbohydrate diet, and vegetarian diet. The PREDIMED (Prevención con Dieta Mediterránea) study provided strong evidence that the Mediterranean diet was an effective dietary model to protect against coronary heart disease.^56^ The randomized trial reported that the incidence of major CVD events was 30% and 28% reductions, respectively, among those assigned to a Mediterranean diet supplemented with extra‐virgin olive oil or nuts than among those assigned to a control (low‐fat) diet.^56^ Low‐carbohydrate diets, defined as 10 ~ 25% total daily energy from carbohydrates (50 ~ 130 g carbohydrate/d), allow the consumption of cardioprotective dietary patterns, including vegetables, fruits, whole grains, nuts, seeds, and legumes.^57^ Medically supervised very‐low‐carbohydrate diets, defined as <10% total daily energy from carbohydrates (<50 g carbohydrate/d), as a means of weight loss and improving CVD risk factors could be beneficial in the short‐term intervention (≤6 months), but it is difficult to maintain.^58^ A ketogenic diet is characterized as replacing the carbohydrate with fat to generate ketosis, the popular version is 5 ~ 10% of total daily energy by carbohydrate and 70 ~ 80% by fat.^59^ Whereas, because of the side effects of the ketogenic diet, include renal stones, dysbiosis, constipation, nutrient deficiencies, keto flu, cardiomyopathy, and decreased bone strength, the beneficial effects of ketogenic diet such as weight loss and glycemic control tend to be difficult to adhere.^60^ After all, the diet guidelines developed in western populations may not fit for Chinese people. We have proposed a traditional Jiangnan diet with considering the dietary habits of the Chinese people, which plays a part in the prevention of cardiometabolic diseases and is cost and taste sustainable.^61^

#### 3.1.5 Sleep disorder

Sleep is one of the most important pieces of human health and sleep deprivation increases the risk of CVD by influencing metabolisms, such as energy expenditure reduction, appetite up‐regulation, and glucose metabolism alteration. Longer sleep duration is also associated with higher CVD risk.^62^ Sleep deprivation induces early damage to vascular structure and function and boosts the activity of the sympathetic nervous system and therefore stimulates the renin‐angiotensin‐aldosterone system to increase catecholamines, further constricts blood vessels, and triggers the onset of hypertension.^63^ Moreover, experimental evidence corroborating harmful cardiovascular effects of excessive sleep duration appeals for more investigation. Therefore, the relationship between long sleep duration and CVD is still a controversy. Longer sleep duration may be a manifestation of poor health, even being considered secondary to undiagnosed illnesses.^64^ Sleep‐disordered breathing, which disrupts the temporal congruence of cardiovascular circadian oscillations, can exert acute and slowly progressive adverse effects on the heart, brain, and circulatory and metabolic function. However, it is an essential thing to notice that the sleep duration assessment acquired through a questionnaire is just a crude measurement of overall sleep. Using wearable devices to assess the sleep quality and quantity including sleep efficiency, sleep fragmentation, and slow‐wave sleep is more accurate to estimate the associations between sleep behavior and CVD risk.

### 3.2 Metabolic risk factors

Obesity, hyperlipidemia, hypertension, diabetes, and other metabolic factors have a pathological role in causing CVD through increased oxidative stress, myocardial fibrosis, myocytes hypertrophy, apoptosis/necrosis of cells, progressive accumulation of senescent cardiac stem cell, and arterial stiffness.^65^, ^66^ Overall, the wide margin of the increase in the morbidity of metabolic risk factors foretells a substantial increase in CVD occurrence in the next decades. There is much to be done regarding interventions at a population altitude and treatments at an individual dimension to control metabolic risk factors.

#### 3.2.1 Obesity

Obesity is considered as the key driver of cardiometabolic comorbidities affecting essentially every organ system and is an appropriate target for primary prevention efforts. Childhood obesity also increases the lifetime burden of CVD morbidity and mortality.^67^ Body fat distribution is a key factor in determining associated health risks. One study using dual‐energy X‐ray absorptiometry assessed regional body fat deposits in postmenopausal women. However, trunk fat provided an increased risk, whereas leg fat decreased cardiovascular risk.^68^ It is widely known that Asians tend to have more visceral fat at lower body mass index levels. Our recent study showed that Chinese adults were more susceptible to the effects of overall obesity and fat distribution on metabolic risk factors compared with other racial/ethnic populations.^69^ Treatment for obesity by using pharmacotherapy and bariatric procedures, adjunctive to complementary lifestyle modification, is associated with effective improvements in CVD risk factors.^70^

#### 3.2.2 Hyperlipidemia

Hyperlipidemia, in particularly elevated LDL‐cholesterol, is extremely common throughout the world. Elevated LDL‐cholesterol was one of the most harmful factors that increase the risk for the development of atherosclerotic plaques and subsequent vascular disease.^71^ LDL‐cholesterol was the strongest modifiable factor associated with atherosclerosis even when all other risk factors were at optimal levels.^72^ All patients diagnosed with hyperlipidemia should comprehend the adverse impact of the disease on their organs and the risks that it may pose, and they would benefit from aggressive medical treatment in addition to diet and lifestyle modifications. There are several medicines to control and manage hyperlipidemia, including proprotein convertase subtilisin/Kexin type 9 (PCSK9) inhibitors, statins, gemcabene, and ezetimibe. When prescribed medications, patients should also be educated on medication compliance, side effects, and the overall risks versus benefits of the medications prescribed.^73^

#### 3.2.3 Hypertension

Hypertension is a leading cause of CVD.^74^ Low awareness in hypertensive patients and the low control rate in treated hypertensive patients are the two major barriers to hypertension control in China. China has steadily promoted the hierarchical medical system, national demonstration areas for chronic disease management, and family doctors to enhance the quality of care over the years. However, the control rates in China were still far from optimal compared with that in developed countries.^75^ BP is affected by nutritional, environmental, and behavioral factors throughout the life course.^76^ There are still controversies about the initiation of treatment and therapeutic target. Many debates are concentrating on the benefits and risks of intensive BP lowering in subgroup populations, such as patients with old age or diabetes. A clinical trial observed that treatment with a systolic BP target of 110 to less than 130 mm Hg resulted in a lower incidence of cardiovascular events compared with a target of 130 to less than 150 mm Hg among the hypertensive patients aged 60 to 80 years old.^77^ Nonpharmacological therapy is also preferred for adults with elevated BP and appropriate first‐line therapy for adults with systolic BP/diastolic BP in 130 ~ 139/80 ~ 89 mm Hg who have an estimated 10‐year atherosclerotic CVD risk of <10%.^78^

#### 3.2.4 Diabetes

Almost 60% of diagnosed diabetes patients are not adequately managed, thereby increasing the incidence of complications.^79^ Approximately half of deaths of diabetic patients are attributable to CVD.^80^ However, there is a great gap between the demands for diabetes management and numbers of health care providers. Lifestyle care with newer technology, such as web‐ or app‐based health care platforms, provides more accurate and valid ways to deliver intervention.^81^ Several challenges still hinder their widespread adoption, including concerns regarding cost‐effectiveness and patient privacy.^82^ Two classes of antihyperglycemic medications, glucagon‐like peptide‐1 receptor agonists and sodium‐glucose cotransporter 2 inhibitors, have demonstrated advantages in reducing CVD risk among individuals with type 2 diabetes mellitus.^83^, ^84^

#### 3.2.5 Non‐alcoholic fatty liver disease (NAFLD)

Currently, NAFLD has become a prevalent disease among adults but remains often undiagnosed.^85^ A nationwide cohort with 13.6 years of follow‐up observed that Swedish adults with histologically confirmed NAFLD were significantly associated with a nearly 65% increased risk of incident CVD.^86^ There are also debates regarding whether NAFLD is an independent CVD risk factor because of residual confounding by other cardiometabolic risk factors.^87^ In 2020, the terminology of NAFLD was proposed to be replaced by metabolic dysfunction‐associated fatty liver disease (MAFLD).^88^ MAFLD is more inclusive in the etiology of fatty liver diseases than NAFLD and is defined based on evidence of hepatic steatosis and concurrently accompanied by the presence of at least one of the following conditions: overweight/obesity, diabetes, or metabolic dysregulation.^88^ Adults with MAFLD were also associated with increased CVD compared with healthy partners.^89^ Lifestyle modification about healthy eating, regular exercise, and weight loss when needed is the cornerstone of therapy on NAFLD, whereas surgery is possible in only a minority of patients.^90^

#### 3.2.6 Chronic kidney disease (CKD)

CKD is regarded as another major risk factor for CVD but often has a strong interaction with other metabolic risk factors.^91^ The other CVD risk factors also exacerbate the progression of CKD. Dyslipidemia accompanied with CKD can contribute to the inflammatory cascade in kidney failure. Hypertension has a bidirectional relationship with CKD, in that it may be the cause of CKD or, instead, a consequence of it. A nationwide survey reported that about 10.8% of adults are suffering from CKD in China in 2009–2010,^92^ and the proportion of diabetic nephropathy and hypertensive nephropathy has surpassed that of CKD resulting from glomerulonephritis since 2011.^93^ In general, recommendations for the prevention of CVD among patients with CKD are undertaken in individuals with normal kidney function, including treatment of dyslipidemia, hypertension and inflammation, and healthy lifestyle interventions. The Study of Heart and Renal Protection (SHARP) trial provides an assessment of the effect of lowering LDL‐cholesterol levels in patients with CKD.^94^ In SHARP, the patients with CKD were randomized to receive a placebo or simvastatin 20 mg and 10 mg of ezetimibe daily.^94^ The simvastatin and ezetimibe yielded an average LDL‐cholesterol reduction of 33 mg/dl, resulting in a 17% proportional reduction in major atherosclerotic events.^94^

### 3.3 Emerging risk factors

#### 3.3.1 Socioeconomic and psychosocial factors

Socioeconomic and psychosocial factors are a group of heterogeneous, but interrelated factors, which cluster in individuals and groups. Low socioeconomic status, including low financial income, less schooling, lack of social support, inequality in health care access, and poor living environment, is an essential part of the etiology of CVD and affects the delivery of CVD prevention. Psychological risk factors are also regarded as emerging, nontraditional risk factors for CVD. Among those, depression has been considered and even used as a modifiable risk factor in CAD patients by AHA.^95^ Psychosocial stress can originate from major life changes, adverse socioeconomic factors, and chronic psychiatric conditions. Linking psychosocial stress and CVD is likely to be common. However, despite all the attempts to promote and support mental health among CVD patients, depressive disorder is still underrecognized and undertreated, particularly in women.^96^

#### 3.3.2 Environmental pollution

Human environments are complex systems; and their natural, social, and personal domains are highly heterogeneous due to variations in human ecosystems, evolutionary histories, social structures, and individual choices.^97^ It is important to understand how the environmental components interact and how they work individually and collectively to play a vital role in CVD risk. Münzel et al summarized the harmful effects of transportation noise on the cardiovascular system and discussed the mechanistic insights.^98^ Noise is closely related to an impairment of redox balance and vascular function in the brain and cardiovascular system.^98^ Noise has a close association with vascular dysfunction, autonomic imbalance, and metabolic abnormalities, contributing to atherosclerosis progression and increased susceptibility to CVD.^99^

Pesticides, depending on their solubility, undergo degradation into different metabolites but may persist for decades. Pesticide exposures leading to the incident of CVD have been proven to be attributed to organophosphates, organochlorines, and herbicides.^100^, ^101^

Ambient air pollution grabs most of the researchersˈ attention. Ambient air pollution has been associated with measures of subclinical atherosclerosis including carotid‐intimal thickness, aortic atherosclerotic plaques, and the progression of coronary calcium.^102^, ^103^, ^104^ Different types of particulate matter are distinguished from each other in chemical composition from internal or external sources and thus may exert distinct health effects. Fine particulate matter <2.5 mm air pollution is the foremost environmental risk factor contributing to CVD mortality and disability.^105^ Environmental exposure in China lies at the central conflict between rapid economic development against the rising CVD burden. Metals including lead, cadmium, and arsenic, the World Health Organization’s top 10 environmental chemicals of concern, also play a role in the development and progression of CVD.^106^ Taking the necessary integration of public and private activities together into account, policy changes at the local levels are needed to address the need for more stringent pollution standards.

#### 3.3.3 Other emerging risk factors

Other emerging risk factors have also received significant attention recently, including apolipoprotein A, apolipoprotein B, high‐sensitivity C‐reactive protein, homocysteine, interleukin‐1, the density and number of LDL‐cholesterol particles, tissue/tumor necrosis factor‐α, ankle‐brachial index, and coronary artery calcium score,..^107^, ^108^ However, the advantages and disadvantages of putting these risk factors into future cardiovascular risk assessment are uncertain. There are also inadequately powered clinical trials demonstrating the preventive effects of lowering these nontraditional risk factors.^108^

## 4 IMPLICATIONS

**FIGURE 1:**
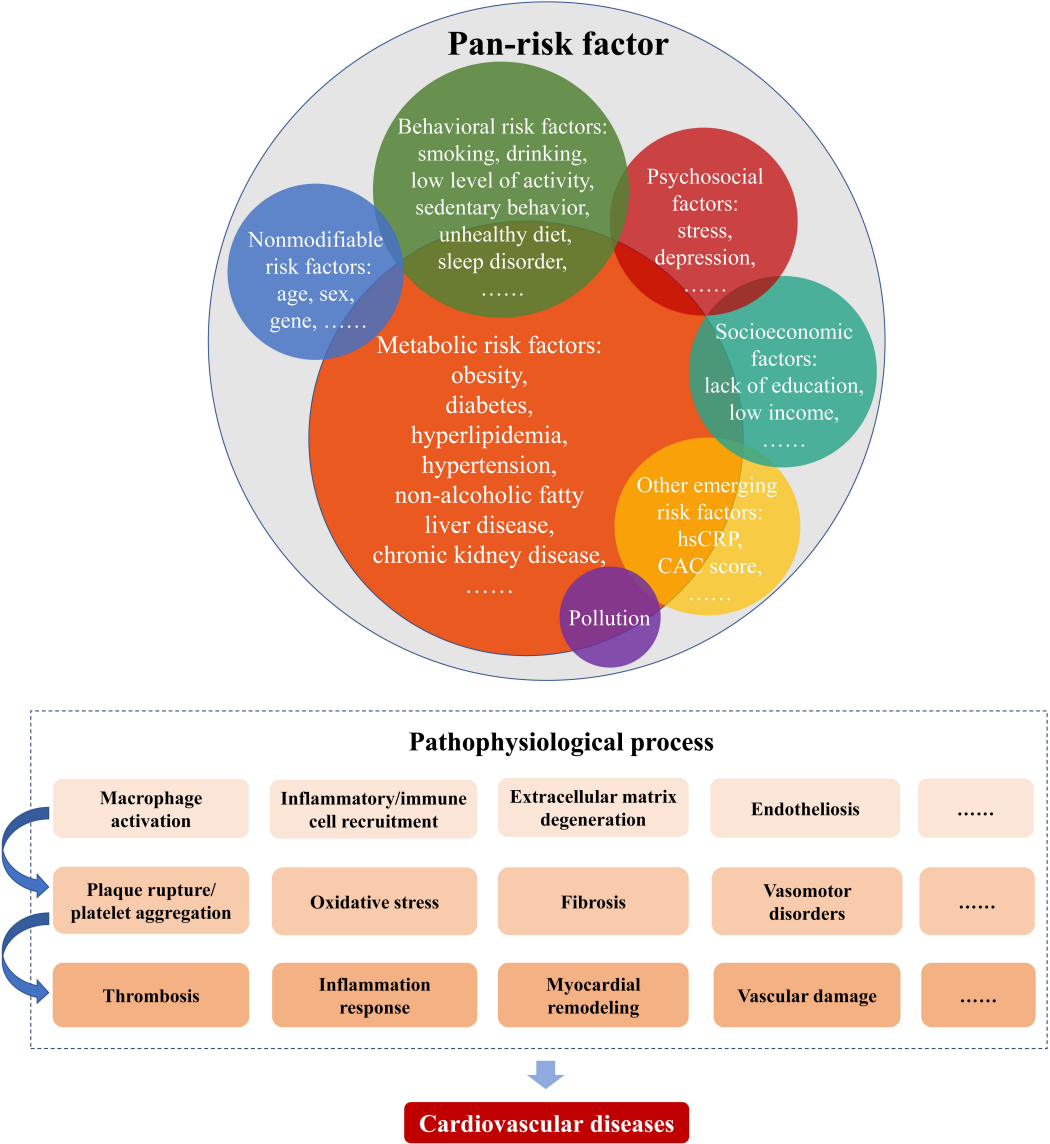
Pan‐risk factor of cardiovascular diseases. CAC, coronary artery calcium; hsCRP, high‐sensitivity C‐reactive protein

The number of CVD cases rose twice since 1990 in China, approaching almost 94 million in 2016.^109^ People may concurrently possess several risk factors and synergically strengthen the adverse effect of multiple risk factors. In the study of Rawshani et al they observed a stepwise increase in the risk of CVD for each additional risk factor among the patients with type 2 diabetes including high glycated hemoglobin, high blood pressure, albuminuria, high LDL‐cholesterol, and smoking.^110^ On the other hand, a meta‐analysis concluded that adherence to several healthy lifestyle behaviors simultaneously including being physically active, eating a healthy diet, maintaining normal weight, low or moderate alcohol consumption, and not currently smoking was associated with 66% reduced CVD compared with adopting none or only one behavior.^111^ By observing the alterations in the spectrum of main risk factors of CVD (including smoking, serum lipids, and blood pressure) in Finland for the past 40 years, it concluded that population‐based primary prevention programs can efficiently reduce the two thirds of coronary heart disease burden and mortality.^112^ Promoting cardiovascular health is a particularly urgent need for China because of the ever‐rising prevalence of these risk factors (Table 1). With more and more risk factors proved to be associated with CVD occurrence, the newly discovered risk factors can be included in the pan‐risk factor to highlight the prevention and control of these factors. The concept of pan‐risk factor can be promoted not only among the physicians and practitioners but also in the general population so as to strengthen the initiative consciousness of self‐monitoring risk factors and maintaining a healthy lifestyle throughout life to minimize the risk of future CVD events (Figure 1). The pan‐risk factor may also contribute to improving the CVD predictive models. Those who are assessed at increased risk of developing future CVD should be taken into consideration to control the pan‐risk factor for lifetime prevention of CVD. By evaluating and controlling the pan‐risk factor of CVD at both the population and individual level, reductions in the incidence of CVD in the Chinese population, especially in those at high risk and who will receive benefit from preventive therapies, are foreseeable.

**TABLE 1:** Summary of the pan‐risk factor

Categories	Summary
Nonmodifiable risk factors	
Age	Age plays a vital part in the deterioration of physiological and cardiovascular functionality.
Sex	Benefiting from the protective effects of estrogen before menopause period, women suffer from CVD several years later than men.^11^
Gene	Genetic background greatly affects CVD risk and hundreds of common genetic variants with small effects are found to be associated with CVD.
Modifiable risk factors	
Lifestyle risk factors	
Smoking	All kinds of smoking exposure, including passive exposure, electronic cigarette smoking, and even a small dose of tobacco smoking, increase risk of CVD.^19^, ^22^
Alcohol drinking	There is still controversy on the protective effects of low or moderate drinking on CVD risk.^25^
Sedentary behavior and physical inactivity	Sedentary behavior and physical inactivity can both independently increase the risk of CVD.
Unhealthy diet	The Dietary Approaches to Stop Hypertension, Mediterranean diet, and low carbohydrate diet are effective dietary models to provide protection against CVD.^56^, ^57^
Sleep disorder	Sleep deprivation not only increases the risk of CVD by influencing metabolism but also induces early damage to vascular structure and function.^63^
Metabolic risk factors	
Obesity	Obesity is the key driver of cardiometabolic comorbidities affecting every organ system.
Hyperlipidemia	Elevated low‐density lipoprotein cholesterol was one of the most common and harmful factors that increase the risk of developing atherosclerotic plaques and subsequent vascular disease.^71^
Hypertension	Low awareness in hypertensive patients and the low control rate in treated hypertensive patients are the two major barriers to hypertension control.^74^
Diabetes	Approximately half deaths of diabetic patients are attributable to CVD.
NAFLD	NAFLD is a “multisystem” disease that not only increases risk of liver‐related complications but also increases risk of developing other metabolic diseases.
CKD	CKD is a major risk factor for CVD but often have a strong interaction with other metabolic risk factors.
Emerging risk factors	
Low socioeconomic status	Low socioeconomic status is an essential part of the etiology of CVD and affects delivery of CVD prevention.
Psychosocial factors	Depression is an emerging risk factor of CVD but still underrecognized and undertreated.^97^
Environmental pollution	Noise and ambient air pollution have a close association with vascular dysfunction and metabolic abnormalities.^99^, ^103^

Abbreviations: CKD, chronic kidney disease; CVD, cardiovascular disease; NAFLD, non‐alcoholic fatty liver disease.

## DISCLOSURE

We declare that we have no conflict of interest.
